# Dysphoric Milk Ejection Reflex: The Psychoneurobiology of the Breastfeeding Experience

**DOI:** 10.3389/fgwh.2021.669826

**Published:** 2021-10-29

**Authors:** Reem Deif, Emily Michelle Burch, Jihan Azar, Nouran Yonis, Macy Abou Gabal, Nabila El Kramani, Duaa DakhlAllah

**Affiliations:** ^1^Institute of Global Health and Human Ecology, School of Sciences and Engineering, The American University in Cairo, Cairo, Egypt; ^2^Department of Biology, School of Sciences and Engineering, The American University in Cairo, Cairo, Egypt

**Keywords:** lactation, breastfeeding, postpartum, maternal, dysphoric milk-ejection reflex

## Abstract

Breastfeeding, given its biochemical and physiological basis, is known for its many benefits for both the lactating mother and the infant. Among the many challenges new breastfeeding mothers experience is the feeling of aversion in response to their newborn's suckling which has been termed dysphoric milk-ejection reflex (D-MER). Characterized by intense feelings of dysphoria which may eventually interfere with the mother's ability to breastfeed regularly, evidence suggests both the neurobiological and psychological basis of D-MER in an attempt to explain its complexity. Biologically, breastfeeding is expressed by the intracerebral release of oxytocin, an increased expression of oxytocin receptors in specific brain regions, increased mesocorticolimbic reward region activation, the secretion of prolactin and possibly the inhibition of dopamine. Hence, different theories explain D-MER in terms of disrupted neurotransmitter and hormonal activity. Breastfeeding has also proven to influence mood and stress reactivity in nursing mothers with a potential link with postpartum depression. Psychological theories attempt to explain D-MER from a sociopsychosexual lense shedding light on the significance of mother-infant attachment, the sexualization of the female body and the motherhood experience as a developmental stage in a woman's lifespan. The aim of this review is to provide a literature update of D-MER incorporating both neurobiological and psychological theories calling for raising awareness about the complexity of breastfeeding and for the need for mother-centered interventions for the management of D-MER and other postpartum-specific conditions.

## Introduction

Breastfeeding, given its biochemical and physiological basis, is known for its many benefits for both the lactating mother and the infant. Despite the recommendations of the WHO and the American Academy of Pediatrics (1) for exclusive breastfeeding till the age of 6 months, percentages of breastfed infants show a decrease over the first months of life (2) and statistics suggest that only 41% of infants between the ages of 0–6 are exclusively breastfed (3). Such rates may reflect the fact that many mothers may initiate breastfeeding, yet stop at a certain point. Research has examined medical and physical challenges in breastfeeding that might predict early cessation, yet little attention has been given to the emotional experiences that might affect the course of breastfeeding. Among the many challenges new breastfeeding mothers experience is the feeling of aversion in response to their newborn's suckling and their need to appropriately position the newborn, nurse and be patient. Clinically, this is referred to as the Dysphoric Milk Ejection Reflex (D-MER) which is characterized by dysphoria starting shortly before ejection of milk and progressing for several minutes. It is likely to recur with each milk ejection response, or in certain cases, only the initial milk ejection response of each feeding session (4). Such sensations create a hollow or churning sensation in the pit of the stomach (5, 6). Symptoms may diminish by 3 months or may persist during the course of breastfeeding (7). Such symptoms have been described from a narrative perspective in different ways; as overwhelming, uncontrollable feelings that have strong influence on the mother, as a sense of obligation toward the baby, as an abnormal experience based on the assumption that a nursing mother should enjoy breastfeeding and finally, as giving a sense of achievement when done. Such feelings may put a mother under psychological pressure and impact her sense of self which may have implications on her relationship with her infant (8). As an underresearched area, the first and only study examining the epidemiology of D-MER was published in 2019 suggesting a prevalence of 9.1% (9).

Symptoms of D-MER may interfere with the mother's ability to maintain an appropriate breastfeeding schedule. For example, women may breastfeed less, and wean earlier (6). Research suggests a prevalence of 20% of postpartum depression among new mothers (10); but whether or not lactation might be a contributing factor is questionable. Although emotional and physical symptoms, such as nausea, associated with D-MER distinguish it from other breast feeding manifestations and postpartum depression (7), symptoms of postpartum depression may contribute to or follow premature weaning in breastfeeding mothers (11), and may overlap with symptoms of other affective problems including dysphoric milk ejection reflex (12).

Given the complex and possibly mutual relationship between breastfeeding and maternal depression, it is likely that breastfeeding complications may influence maternal affect and mood. For instance, Brown et al. (13) observed that breastfeeding cessation in mothers is associated with high depression scores among mothers who quit breastfeeding due to physical difficulties and discomfort while breastfeeding. At 8 weeks postpartum, another research examined breastfeeding difficulties and maternal mood and reported that breastfeeding difficulties were correlated with worse maternal mood, or other comorbid physical problems (14). However, although breastfeeding is linked with maternal mood and postpartum depression, it is hard to ascertain whether the effects are triggered by breastfeeding or maternal mood given the complicated relationship between both. Research findings on the link between breastfeeding and postpartum mental health is still inconsistent. For example, breastfeeding is less prevalent among mothers with depression, it might help prevent postpartum depression (15), but it is still related to postpartum depression in some cases (16). The function of oxytocin is significantly involved, not only in breastfeeding, but also in postnatal depression (17) by reducing neuroendocrine stress signaling and anxiety-related and depressive symptoms (18).

Looking into the history of D-MER, it was first identified by Alia Macrina Heise in 2007. In 2008, Heise created the web domain, www.d-mer.org, to educate the public about this condition and provide support for mothers (19) and in 2010, the first D-MER case study was issued. Two other case analyses were released in 2011 and 2018 (5, 19). Unfortunately, there is not enough evidence confirming D-MER as a clinical phenomenon given the limited case cases and anecdotal data (4). In this regard, the aim of this paper is to provide a literature update on D-MER from a biopsychosocial perspective. A literature search has been performed in PubMed, ScienceDirect, MedLine, and APA PsycArticles, and given the limited literature available on this subject, research needs are also highlighted toward the end of this review.

## Breastfeeding From a Hormonal/Endocrine Perspective

Lactation or lactogenesis is established in two phases. Stage one is known as secretory differentiation and it is followed by stage two or secretory activation (20). The first stage of lactogenesis begins around 15–20 weeks of gestation and is primarily driven by hormones (21). Secretory differentiation can be observed as the breast develops the capacity to synthesize colostrum, breastmilk, and other milk products like lactose, casein, a-lactalbumin, and lactoferrin (20).

The mammary gland is an exocrine gland that produces breast milk in humans. This gland faces a number of developmental changes when preparing for lactation. Gland maturation and alveologenesis are among two of these changes. The formation of alveoli, milk secreting cells, is primarily driven by a hormone called progesterone and this process occurs early on in pregnancy (22). Lactation in alveolar cells is induced by a hormone called prolactin. Mammary glands are sufficiently developed around 20 weeks of gestation by way of both prolactin and progesterone. Following the development of the mammary glands, the accumulation of colostrum begins. Colostrum production is usually initiated about halfway through pregnancy and continues through the third trimester (23). Breast size and volume usually increase as a result of these factors. The amount of change that occurs can vary significantly between mothers. It is important to note that the size of the breast or the increase in volume do not determine the amount of milk that will be synthesized postpartum (20).

Secretory activation begins anywhere from 24 to 72 h after birth and is triggered by the birth of the infant and the delivery of the placenta (20). Women feel increased breast fullness in this second stage. After birth, progesterone levels decrease rapidly and prolactin and oxytocin levels increase (23). Prolactin is able to induce the secretory activity of alveolar cells and promote the release of milk into alveoli and smaller ducts (21). Prolactin responds to nipple stimulation, infant suckling, and expression of breastmilk. Oxytocin triggers the let down reflex or the release of milk when the nipple areola is stimulated (23). Although prolactin and oxytocin are the primary hormones in this process, Insulin, cortisol, and thyroxine are also involved (21).

The first h following the birth of a child is often referred to as the “Magical hour.” This first h can be a strong determinant of breastfeeding success. The reason this hour is so important is because the composition of this first feed will be colostrum since secretory activation, or the arrival of breast milk has not occurred yet. Colostrum is very rich in antibodies and high in nutritional value that is sufficient to meet a newborns' nutritional needs immediately following delivery (21). The “Magical Hour” is more than the introduction of colostrum and breastfeeding alone. It provides a vital time for skin-to-skin contact between the mother and her newborn. It allows the newborn to bond and to learn to utilize their olfactory sense to properly latch onto the mothers breast. Srirama et al. showed that early lactation stimulates the development of more prolactin receptors. The increase in prolactin receptors may enhance the potential for future breast milk production (21).

Hormones are key elements in both secretory differentiation and secretory activation regardless of whether breastfeeding is initiated. Following these two stages, ongoing milk production is also controlled by hormones, however unlike the first two stages it is determined by the removal of milk from the breasts (21). Ongoing milk production is an issue of supply and demand. When the milk has been secreted out of the breast, hormonal signals are sent to the hypothalamus to initiate more milk production. Lactation will continue as long as milk is being secreted and removed from the breast. The breast capacity to store milk can range anywhere from 80 to 600 ml. In breasts that have lower storage capacity, milk synthesis will be a more rapid process when compared to breasts with a larger storage capacity (23). This is to ensure that there is sufficient milk to meet the infant's needs. It is recommended that infants are fed on demand based on their hunger cues. It is estimated that a newborn will feed at least 10–12 times a day (21).

Breast milk provides the appropriate nutrients to an infant based on their developmental stage. Breast milk also provides proteins, growth factors, antimicrobial peptides and proteins to support an infant's immune system and gastrointestinal system (24). Exclusive breast feeding is recommended for the first 6 months of life (25). Introducing formula early on can adversely affect breast milk production and can lead to an increased risk of breastfeeding cessation. Formula should be introduced only if there is a medical need indicating that supplementation is required. This will optimize milk supply and increase the chances of exclusive breastfeeding until it is time for the introduction of complementary foods (21).

The Milk Ejection Reflex (MER) is an essential element in milk production. MER helps to both establish and maintain milk production. MER may also be referred to as the “let down reflex.” This is a neuro-endocrine reflex that is brought on by the stimulation of the nipple and areola, the circular area around the nipple (21). The negative pressure related to the infants sucking results in the 4th intercostal nerve sending signals to the hypothalamus to release oxytocin. Oxytocin is released from the posterior pituitary gland. The release of oxytocin can be enhanced by a variety of factors, such as, hearing the baby cry, thinking about the baby or preparing to breastfeed. The release of oxytocin can also be inhibited by fear, pain, embarrassment or anxiety of the mother (21).

## The Psychology of Breastfeeding

On the healthy end of the spectrum, breastfeeding is believed to foster maternal responsiveness and healthy mother-child attachment (26, 27). For instance, breastfeeding mothers hold their babies more, are more receptive, and spend more time nursing in mutual gaze than bottle-feeding mothers (28). On the neural level, brain mapping shows higher brain activity in different limbic brain regions in breastfeeding mothers when responding to the infant's cries (29). Breastfeeding has also been shown to influence mood and stress reactivity in different ways. For example, in comparison to formula-feeding mothers, breastfeeding mothers may show less agitation, depressive disposition, stress (30), better modulation of cardiac vagal tone, lowered blood pressure, and decreased heart rate, and an overall relaxed and non-anxious physiological state (31). Additionally, breastfeeding has shown to be associated with a diminished reaction to cortisol when dealing with social tension (32) and longer and higher quality sleep cycles (33). In terms of social cognition, breastfeeding may positively affect a mother's reaction to others as suggested by research showing an association between extended breastfeeding durations and positive responses to happy facial expressions and less responsiveness to angry ones (34).

From a neuropsychological perspective, it can be confidently claimed that oxytocin does not only play a role in the process of milk production, but also in the emotional attachment between the mother and the newborn during lactation. This also confirms the link between breastfeeding and low cortisol levels in order to promote low-stress interaction between the mother and the infant (35).

In order to understand the psychopathology of D-MER, motherhood should first be conceptualized from a psychological perspective as an independent developmental stage in a woman's lifespan. Breastfeeding in itself can be regarded as a part of the emerging motherhood identity. This also means that some lactating mothers may not have a personal meaning of breastfeeding and may, therefore, discontinue breastfeeding or introduce formula at an early age. Such identity problems may also affect maternal mental health and may result in less pleasurable experiences of breastfeeding (36).

Different psychological factors may come into play when a mother makes the decision of whether or not to breastfeed (37). One systematic review highlighted the effect of self-efficacy, postpartum mental health, social support, intention and attitudes toward breastfeeding as psychological predictors of exclusive breastfeeding duration (38). Other concerns which might interfere with the mother's decision to breastfeed include the mother's feelings of incompetence, anxiety about the baby's nutrition and/or embarrassment about her bodily exposure (39), maternal sense of autonomy (40), and the mother's perception of the social context needs and the needs of others (41). Exposure to intimate partner violence has also shown to increase the risk of breastfeeding avoidance by double (42).

The sexualization of a woman's breasts besides the need to expose them to breastfeed, depending on the level of privacy provided, may create a sense of confusion for the nursing mother. That is why embarrassment and high levels of modesty may interfere with a mother's willingness to breastfeed (43). Supporting such a hypothesis, more recent research has examined self-objectification and “reproductive shame” associated with different reproductive roles including breastfeeding (44). On the other hand, women who prefer not to breastfeed may have less positive attitudes toward sex as suggested by Sears et al. (45).

One study aiming to explore the mother's narratives of their negative embodied emotional sensations while breastfeeding using interpretative phenomenological analysis suggests the influence of breastfeeding on initiating conflicting thoughts and emotions which in itself can impact the mother's self image and the way they relate to their newborns. Three themes were identified; breastfeeding as an unexpected trigger of intense embodied emotional sensations incongruent with view of self; fulfilling maternal expectation and maintaining closeness with the child; and making sense of embodied emotional sensations essential to acceptance and coping. Put together, such findings call for raising awareness about the complexity of the breastfeeding experience and for the need for mother-centered interventions (6).

## The Neurobiology of Breastfeeding

Different hypotheses of mechanisms of action underlying dysphoria have been proposed, but dopamine has been the most probable candidate (19). The secretion of prolactin, which also happens during lactation, is dependent on the inhibition of dopamine (5). Pseudoephedrine, which stops milk production, was seen to eliminate D-MER without acting on oxytocin, but rather by reducing levels of prolactin. Whether this occurs by increasing dopamine levels is unknown (19). Dopamine is thought to be more abrupt in rising and falling during breastfeeding, therefore it might correlate more with D-MER (19). Transient reduction in dopamine in the hypophyseal portal veins has been observed resulting in elevated prolactin levels (19).

Breastfeeding is generally associated with the upregulation of endogenous oxytocin levels. Research also shows that mothers' genetic variance in oxytocin gene influences the pace at which cortisol declines throughout breastfeeding sessions. Similar reductions in infants were also observed (28). Research has shown that intranasal administration of oxytocin was shown to reduce anxiety in non-breastfeeding contexts (46, 47). Such findings might be implicated in relation to reducing aversion in cases of D-MER by regulating levels of oxytocin.

During breastfeeding, there is an increase in intracerebral release of oxytocin and the expression of oxytocin receptors in specific brain locations. Nipple stimulation is also believed to stimulate oxytocin production. Higher circulating oxytocin levels correlate with increased mesocorticolimbic reward region activation (48). Hence, reduction in oxytocin might be implicated in the dysphoric symptoms in D-MER. Dopamine was also found to stimulate the release of oxytocin in rats while lowering it in others. However, the neurotransmitter release pattern is difficult to determine since the brain is not as easily penetrated as the blood (19).

Glutamate might also play a role in the release of oxytocin. The effect of glutamate on dopamine is still rather unknown. Emotions, ranging from desire to disgust, triggered by dopamine are mainly due to the effect of glutamate on different locations in the brain. To strengthen the hypothesis that dopamine is responsible for the dysphoria caused during breastfeeding, is the fact that dopamine reuptake inhibitors such as bupropion, which increases the availability of dopamine, resulted in the elimination of D-MER symptoms in one woman. Herbals such as Rhodiola rosea or golden root acts as monoamine oxidase inhibitors which inhibits the breakdown of dopamine and other neurotransmitters, and may improve symptoms of D-MER in nursing mothers (19).

## Discussion and Implications

Due to poor public awareness of D-MER and the scarcity of evidence-based literature, many mothers may mistake D-MER for postpartum depression especially given its atypical symptomatic manifestations, and lactation practitioners and health care providers may also barely recognize D-MER (49). Another challenge in the management of D-MER is that mental health professionals may lack knowledge about lactation or training in lactation management (12). This makes it necessary to educate mothers because educated mothers are usually better at handling postpartum situations if they are prepared in advance. Given the variety of emotions experienced by breastfeeding mothers with D-MER, the fact that symptoms may persist up until weaning and the potential risk of suicidality, professional intervention should always be recommended and tailored based on the mother's needs and the severity of symptoms (19). Generally, research shows the positive effects of breastfeeding support on increasing the overall breastfeeding duration (50) and the effectiveness of relaxation techniques on improving maternal and newborn outcomes (51). Other psychosocial-based breastfeeding promotion programs exist, yet the extent to which such programs are evidence-based and effective is unclear (52). Although not specific to D-MER, such findings may give hope to the introduction of more specialized interventions for depending on the severity of D-MER symptoms, risk factors or comorbid postpartum mental health problems. Preliminary recommendations highlight the need to alleviate the stress that is triggered during breastfeeding through creating a safe environment for the lactating mother (53). Other forms of psychological management which have been suggested include lifestyle adjustments (54) as mothers may still perceive themselves as “bad mothers” blaming themselves for experiencing such emotions.

## Conclusion

Dysphoric milk ejection reflex is a common, yet not a commonly researched, condition among breastfeeding mothers that is often confused with other postpartum conditions. Emotional and physical symptoms may help distinguish D-MER from other disorders and should be considered for the effective management of this condition. Preliminary research shows the involvement of different hormonal, neurobiological and psychological mechanisms in the pathology of D-MER paving the way for future research to attempt to understand overlaps between different mechanisms and to suggest evidence-based prevention and treatment protocols.

The authors are aware of the limitations of this review given the lack of experimental and epidemiological research in the area of D-MER. A limited number of theoretical research was available through different scholarly databases and all were included in this review. The foundations have been presented to raise awareness about the complexity of breastfeeding, as a physiological function, health behavior and emotional experience, and to encourage more evidence-based research in this area.

## Author Contributions

RD has put the outline for this review and contributed to the sections of the psychological aspects of D-MER with NE. EB focused on the part of breastfeeding from a hormonal/endocrine perspective. JA, MA, and NY worked on the neurobiology of breastfeeding and D-MER specifically. DD worked on revising the outline of the paper and editing the sections accordingly, adding recommendations, and proofreading the paper. All authors contributed to the article and approved the submitted version.

## Conflict of Interest

The authors declare that the research was conducted in the absence of any commercial or financial relationships that could be construed as a potential conflict of interest.

## Publisher's Note

All claims expressed in this article are solely those of the authors and do not necessarily represent those of their affiliated organizations, or those of the publisher, the editors and the reviewers. Any product that may be evaluated in this article, or claim that may be made by its manufacturer, is not guaranteed or endorsed by the publisher.
